# Effect of taurine in muscle damage markers and inflammatory cytokines in running exercise

**DOI:** 10.3389/fphys.2022.1008060

**Published:** 2022-09-13

**Authors:** Yucong Wang, Tao Xu, Hui Zhao, Chunxiao Gu, Zhongzheng Li

**Affiliations:** Department of Joint Surgery, Ningbo NO9 Hospital, Ningbo, China

**Keywords:** taurine, running, exercise, muscle damage, inflammation

## Abstract

This study aimed to investigate the effect of taurine on muscle damage markers and inflammatory markers in the running. For that, ten healthy volunteers participated in this study (mean ± SEM; age 24 ± 1 year, body mass 72.2 ± 4.89 kg, height 174.03 ± 2.85 cm, and BMI 23.83 ± 1.27). The running exercise was performed for 5 km, and blood was taken pre-exercise and pre-exercise + tau and post-exercise and post-exercise + tau for biochemical assessment. We assessed serum creatine kinase (CK), CK isoenzyme, Lactate dehydrogenase (LDH), aspartate transaminase (AST), tumor necrosis factor-alpha (TNF-alpha), and interleukin-6 (IL-6). CK level was not significantly different in the control and taurine (tau) administrated groups. However, creatine kinase isoenzyme was decreased in the pre-exercise + tau group when compared to the post-exercise + tau group. AST level was increased significantly in the post-exercise compared to the post-exercise + tau group. There was no significant difference observed in the LDH level in both post-exercise and post-exercise + tau. TNF-alpha level was not also significantly different in both post-exercise and post-exercise + tau. However, IL-6 was decreased in the post-exercise + tau when compared to the post-exercise group. In conclusion, we observed that taurine decreases the inflammatory response by decreasing IL-6 and AST, suggesting the role of taurine in regulating inflammatory response could help to increase running performance.

## Introduction

It is obvious that any form of physical activity induces health benefits. However, identifying low-cost methods and increasing physical performance is still debatable. In many types, running is the most feasible and readily available exercise option for many people. Indeed, running and jogging contributions increased among the people (Ooms et al., 2013; Franken et al., 2022). However, improving running performance, such as performing faster and longer, requires a specific strategy. Recommending supplements before the exercise may increase physical performance. Many so-called energy drinks are being prescribed in the market the name of claiming that they can increase endurance and performance (Breda et al., 2014; Alsunni, 2015; Visram et al., 2016). However, less research has been reported on producing benefits, mainly when these supplements are provided before the exercise. Usually, these energy drinks contain several compounds, including caffeine, vitamins, and taurine, which specifically interferes with the health benefits in various ways, such as regulating cellular functions.

Energy drinks during exercise have been repeatedly reported to delay muscle fatigue and improve physical performance. However, these energy drinks have high calorific value, which influences the important active compound’s bioavailability and their biological properties in it (Vist and Maughan 1995). Thus, administering a drink with low calorific values or proper composition of such active compounds could overcome exercise-induced muscle damage and improve exercise performance. Taurine is one of the important compounds in red bull and in skeletal muscle, heart, and other tissues at a low level (Gutiérrez-Hellín and Varillas-Delgado., 2021). Studies reported that taurine regulates osmolarity, membrane stabilization, and calcium kinetics, improving systemic anti-inflammatory response and total antioxidant capacity (De Luca et al., 2015). These properties could directly promote physical performance and decrease muscle fatigue. In contrast, a study reported that chronic supplementation of taurine (5 g/day for a week) did not improve exercise performance (Galloway et al., 2008). Furthermore, chronic ingestion of taurine had no effect on energy compounds such as ATP and glycogen, along with the metabolic response to moderate exercise (Galloway et al., 2008). This demand is revisiting the effect of taurine in improving exercise performance. Although the presence of taurine is high in skeletal muscle, its role in regulating exercise-induced muscle damage, inflammatory response, and oxidative stress are still inconclusive. Therefore, this study aimed to investigate whether taurine improves running performance by decreasing inflammatory response and muscle damage.

## Materials and methods

### Trail design and participants

Ten recreational runners were recruited from Ningbo city. Participants were informed before the experiments started, obtained their concerns in writing, and explained the risks orally linked with this experiment, and all participants agreed to the experiment. All the Participant’s characterization is given in Table 1. Inclusion criteria were: subjects who had completed their running exercise up to a 5-km distance in under 35 min. All the participants were asked to refrain from the use of any ergogenic aids for at least a month before the experiment started. Also, they were asked not to perform any exercise before testing. Also, they were asked not to consume energy drinks and alcohol for 48 h before running exercise started. The exclusion criteria were that participants who had any medical issues on the day of the running exercise were excluded.

**TABLE 1:** Characterization of the participants.

	Experimental Group *n* = 5	Control Group *n* = 5	*P*
Height (cm)	174.03 ± 2.85	173.27 ± 3.32	0.609
Body mass (kg)	72.2 ± 4.89	70.6 ± 4.82	0.494
BMI (kg/m^2^)	23.83 ± 1.27	23.51 ± 1.31	0.588

### Supplementation

The selected participants were asked to follow any one of the treatments as follows; participants consumed 250 ml of a commercially available energy drink (Red Bull^®^), which was purchased at Yongxin supermarket, Fenghua Road, Jiangbei District, Ningbo, Zhejiang Province. A total of 250 ml of red bull contains Taurine 125 mg, caffeine 50 mg, Inositol 50 mg, lysine 50 mg, nicotinamide 10 mg, vitamin B 61 mg, and vitamin B123 μg. The placebo condition consisted of 250 ml of water.

### Running condition

**FIGURE 1:**
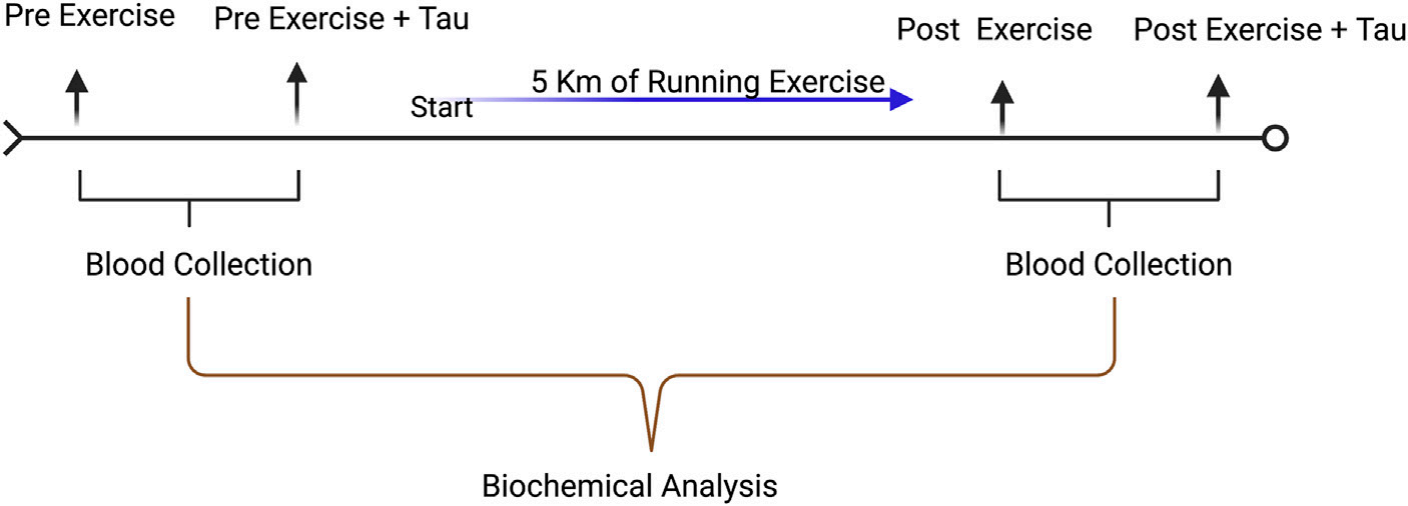
Timeline illustration of experimental design. Blood collection was carried out before and after the running exercise with the control group and supplementation group.

The 5 km of running was performed in April 2022 in Ningbo city around the city in dry weather with pleasant conditions (30–35°C, light wind). All the participants were informed about the running protocol as mentioned above. The runners were allowed only one time to consume either 250 ml of water or Red bull. The running exercise timeline is given in Figure 1.

### Blood sample

Blood samples (10 ml) were drawn prior to the exercise and after the exercise. The blood samples were drawn from the antecubital vein, collected in vacutainers without additives, and centrifuged at 1,500 rpm for 10 min at 4°C. Aliquots of washed/lysed red blood cells and serum samples were stored at −70°C until biochemical assays were performed.

### AST, LDH, CK, and CK-isoenzyme assessments

AST, LDH, CK, and CK-isoenzyme levels were measured from the serum using a commercial kit (Anhui Yi Pu Nuo Kang Biotechnology Co. Ltd.) according to the instructions provided by the manufacturer. The results of AST, LDH, CK, and CK-isoenzymes were expressed as U/L.

### Measurement of TNF-alpha and IL-6

The serum levels of TNF- alpha and IL-6 were determined by commercially available ELISA kits (R&D Systems, Minneapolis, MN, United States)

### Statistical analysis

Data were expressed as means ± standard deviation (SD). Kolmogorov-Smirnov test was used to confirm the normal distribution of the values of all analyzed parameters. The significance of differences was calculated using one-way ANOVA, followed by Bonferroni post hoc tests, as indicated in the legends of the figures. Statistical significance was set at *p* ≤ 0.05. Graph Pad Prism software version 9 was used for all the analysis.

## Results

**FIGURE 2:**
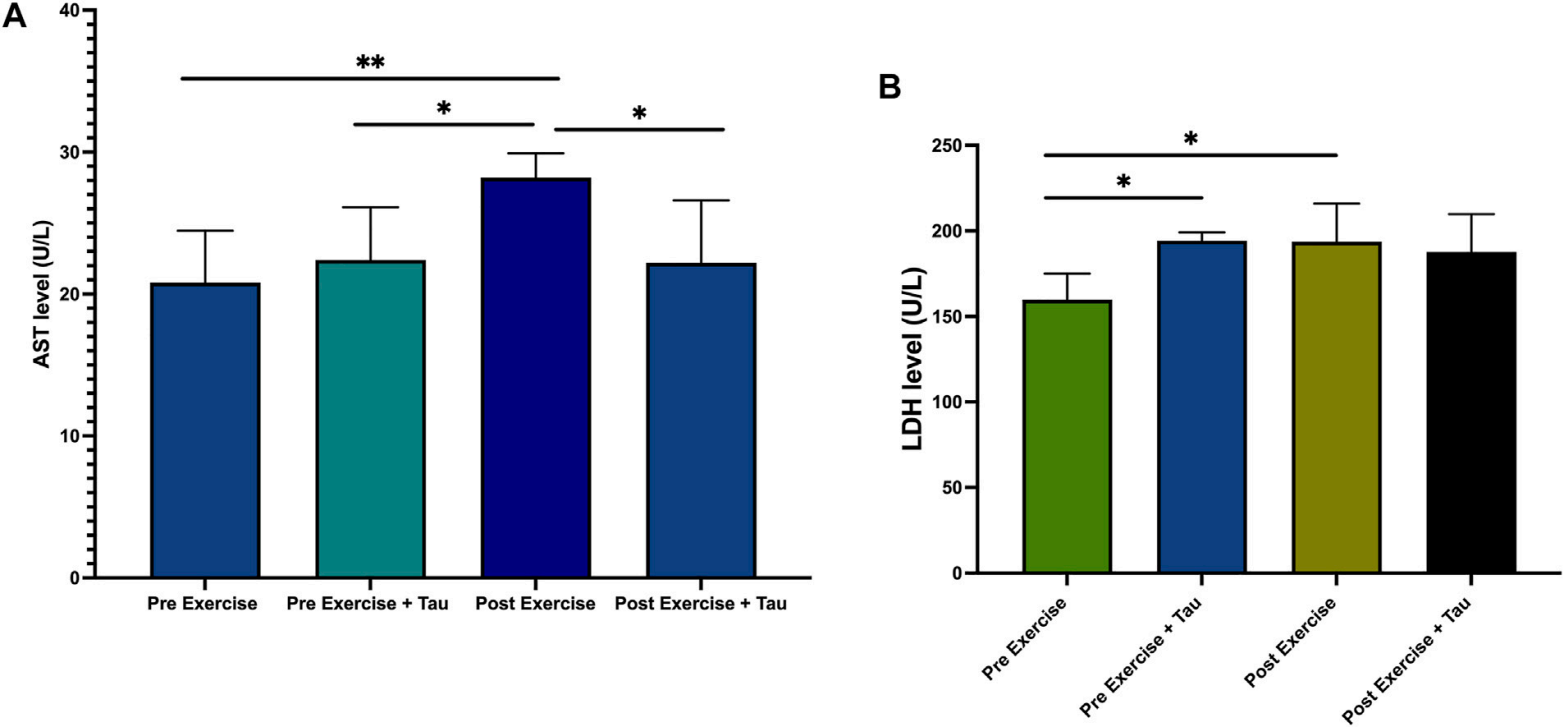
Effect of taurine (tau) on running exercise. AST level decreased in pre-exercise significantly when compared to post-exercise (**p < 0.05). AST level decreased in post-exercise + tau when compared to post-exercise (*p < 0.05) (Figure 2A). No changes were obtained in the LDH level of both postexercise and post-exercise + tau (Figure 2B).

AST level was increased significantly in post-running exercise when compared to a pre-exercise, while AST level was decreased significantly in post-exercise + tau when compared to post-exercise. This study observed that there was no significant difference between the post-exercise and post-exercise + tau with regard to LDH level, but LDH level was increased in the post-exercise compared to the pre-exercise group (Figures 2A,B).

**FIGURE 3:**
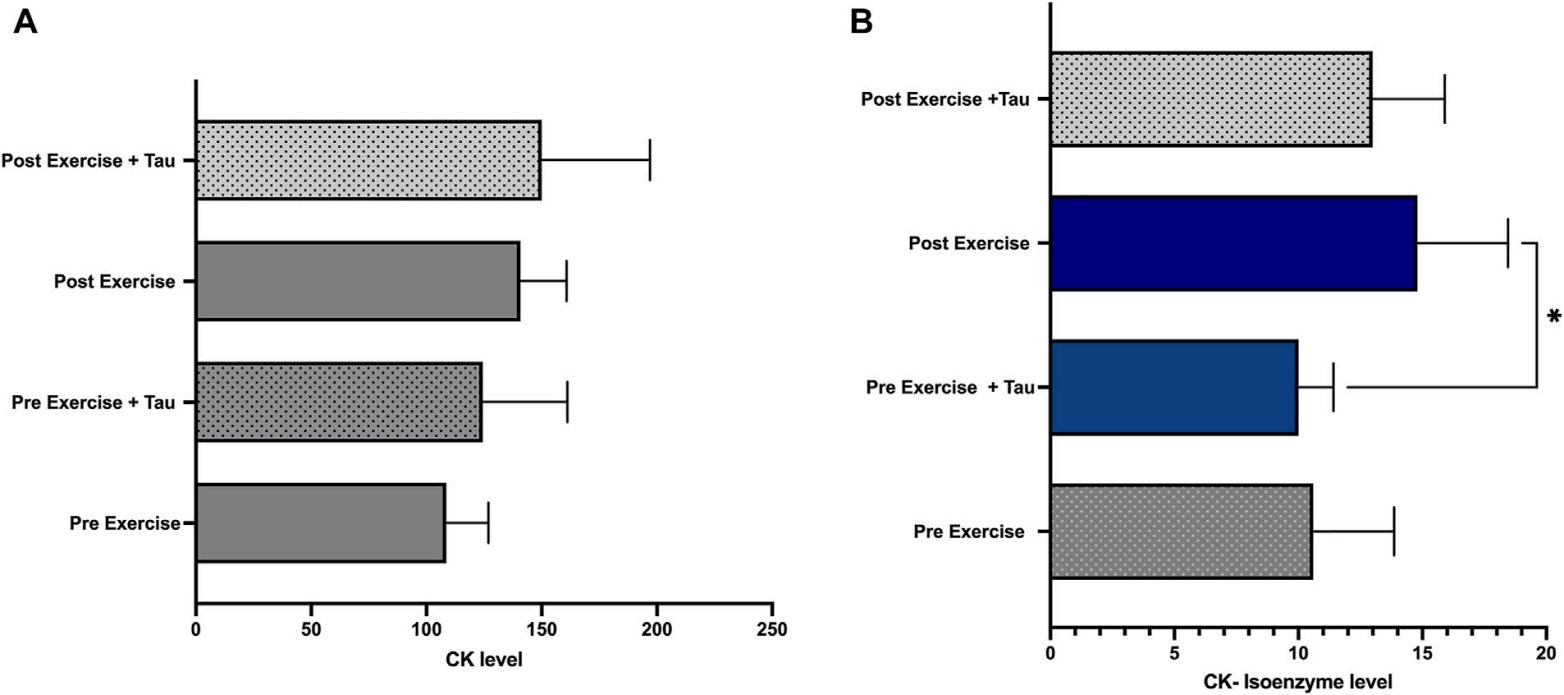
Effect of taurine on CK and CK-isoenzyme levels. No significant changes were observed in the total CK level of all the groups (Figure 3A), while CKisoenzyme level was decreased significantly in pre-exercise + tau when compared to post-exercise (*p < 0.05) (Figure 3B).

This study observed no significant difference in the total CK level between all the groups. However, CK-isoenzyme level was significantly decreased in pre-exercise + tau when compared to the post-exercise group (Figures 3A,B).

**FIGURE 4:**
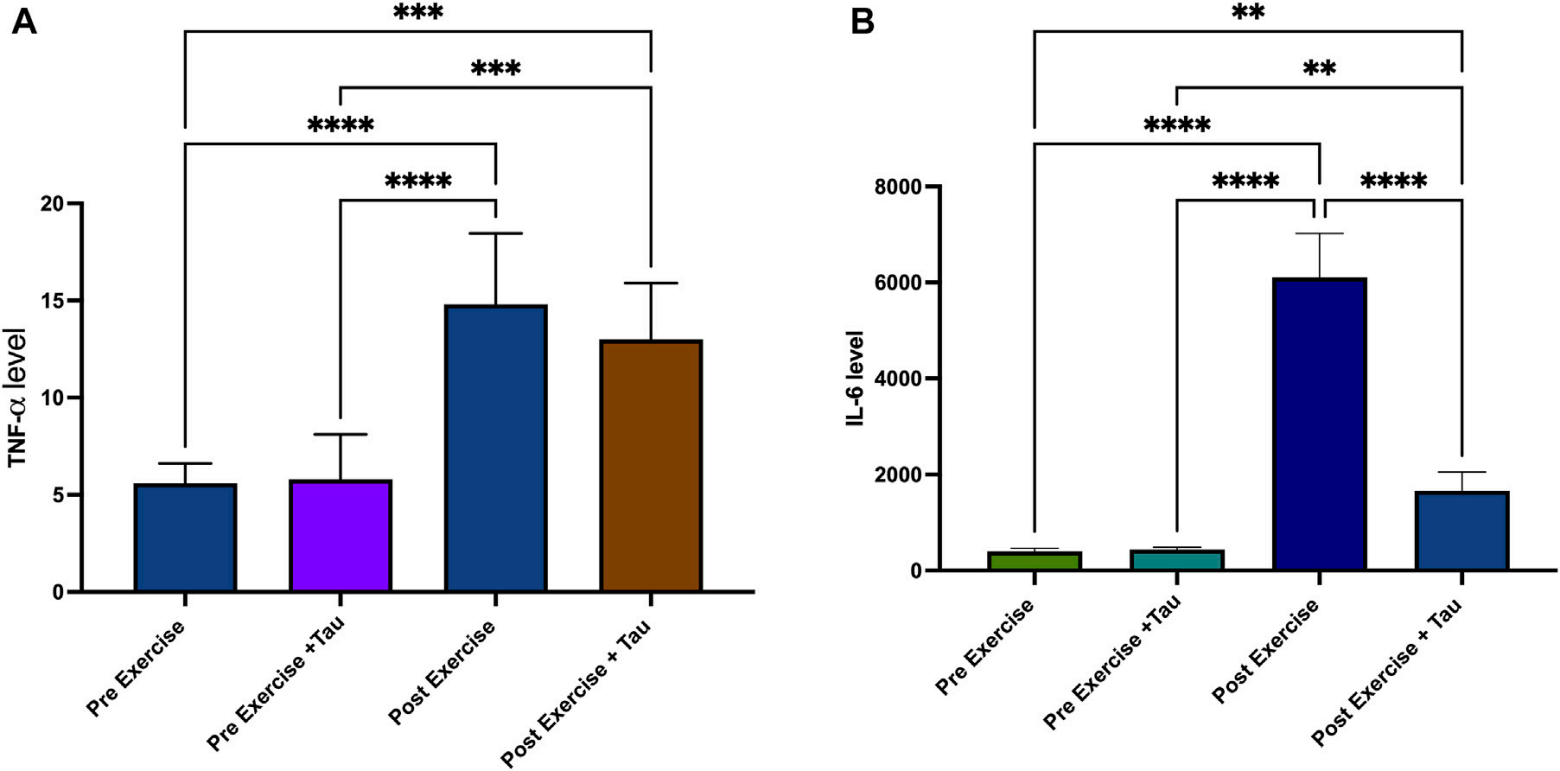
Effect of taurine on TNF-alpha and IL-6 levels. TNF-alpha level was increased in post-exercise and post-exercise + tau significantly when compared to pre-exercise and pre-exercise + tau (***p < 0.05). TNF-alpha was decreased in post-exercise + tau when compared to pre-exercise (***p < 0.05). IL-6 level was increased in post-exercise and post-exercise + tau when compared to pre-exercise and pre-exercise + tau (****p <0.05) (Figure 4A), while IL-6 level was decreased significantly when compared to post-exercise (****p < 0.05) (Figure 4B).

This study selected two inflammatory markers to find the effect of tau in running exercise. TNF-alpha level was not significantly decreased in post-exercise + tau compared to post-exercise. In contrast, it was increased in the post-exercise + tau. IL-6 level decreased in the post-exercise + tau group compared to the post-exercise group. However, both TNF-alpha and IL-6 levels were decreased in the pre-exercise and pre-exercise + tau groups when compared to post-exercise and post-exercise + tau groups (Figures 4A,B).

## Discussion

Transaminase levels are misspelled to interpret liver function tests (Pavletic and Pao, 2015). Although it is present in the liver, it can also present in other tissues such as muscles and the heart. Indeed, muscle has a higher level of AST and ALT as it is a larger tissue mass. Therefore, elevated level of AST and ALT is associated with muscle damage and muscle disorder. Consequently, decrease the exercise-induced benefits and its performance. Studies observed that increased physical activity is linked with an increase in AST and ALT levels. This may be due to the dysregulation of inflammatory cascades and oxidative stress. The present study showed that AST level was decreased in the post-exercise + tau when compared to the pre-exercise group and post-exercise, proposing the role of taurine in regulating AST. Running induced inflammatory response could alter the AST level in the blood, and taurine`s anti-inflammatory property could revert AST to a normal level. Animal models showed that taurine decreases the AST and ALT by regulating inflammatory response and oxidative stress (Zhang et al., 2014; Abdel-Moneim et al., 2015; Liu et al., 2017).

Although studies reported that alteration in the CK and LDH level indicates muscle damage, the increase of CK and LDH after running has been suggested to measure the recovery capacity of athletes (Brancaccio et al., 2006; Callegari et al., 2017). Therefore, this study aimed to investigate whether taurine decreases these parameters to improve the recovery capacity of running participants. The role of taurine in influencing CK and LDH has been reported unequivocally. For example, a decrease in taurine contributes to reducing ATP biosynthesis (Schaffer and Kim, 2018). This could influence the level of CK in the blood and muscles. However, our study observed that taurine had no role or limited role in ATP depletion, which is confirmed with no significant differences in the CK and CK isoenzyme levels in the post-exercise and post-exercise + tau. This may be due to the finishing time and intensity of running exercise.

Consequently, it affects the release of CK into the blood. Studies reported that marathon completion time is positively linked with the levels of CK (Siegel et al., 1980; Stansbie et al., 1983). Next, this study observed no significant difference in LDH in pre- and post-exercise groups. This may be due to differences in the LDH isoenzymes distribution among the participants. This has been reported with athletes and nonathletes who had fluctuated LDH isoenzymes patterns. Galan et al. reported that taurine supplementation did not alter the LDH and CK levels during 8 weeks of exercise (Galan et al., 2018). Following these studies, our study did not increase both LDH and CK levels. This may be due to the fluctuations in the intensity and finishing time, which limit the LDH and CK level increment (Galan et al., 2018).

Cytokine response to exercise is linked with the amount of damage that is occurred in the muscle due to running exercise (Pedersen et al., 2001). Studies showed that IL-6 increases in response to exercise-induced muscle damage (Nielsen et al., 1996). This could be reverted using supplements, including vitamins and antioxidants. For example, De Carvalho showed that taurine reduced the IL-6 level in exercised conditions and increased the anti-inflammatory cytokines (De Carvalho et al., 2021). Studies have already reported the anti-inflammatory properties of taurine, which may be related to its neutralizing capacity of HOCl, an oxidant that can overwhelm the redox capacity of the system. Taurine reacts with HOCl to generate TauCl (taurine chloramine), which can regulate cytokine production such as IL-6 and TNF- alpha. Also, it can be able to modulate inflammasome signaling. This study showed that IL-6 was decreased in post-exercise + tau when compared to post-exercise. However, there was no change observed in the TNF-alpha level. Rosa et al. reported that taurine did not increase the TNF- alpha level in obese conditions (Rosa et al., 2014). De Carvalho showed a decrease in IL-6 in taurine and exercised obese women (De Carvalho et al., 2021). To corroborate these studies, our study did not decrease the TNF-alpha level in the post-exercise + tau. This may be due to a decrease in the blood flow during running, which could aggravate the inflammatory response in the running participants. Studies reported that marathon running decreases the blood flow to the splanchnic organs and induces ischemic events (Bagby et al., 1996; Camus et al., 1997). Taken together, taurine decreased the inflammatory response by decreasing IL-6 and AST.

### Study limitation

This study has some limitations as follows: we study used commercially available red bull, which not only contains taurine but also contains other ingredients such as caffeine, lysine, and vitamins, which might influence altering the muscle damage markers and inflammatory cytokines. Also, we study used a minimum sample, which prevents the complete recommendation of the ingestion of red bull.

## Conclusion

This study showed that taurine reduces the inflammatory parameters (TNF-alpha and IL-6) and AST in 5 km running without altering the level of other muscle damage markers such as CK and LDH, proposing the role of taurine in regulating the inflammatory response during running without affecting muscle damage markers. Consequently, taurine improves the running exercise performance. However, a low number of samples in this study limited the complete benefits of taurine. Therefore, further studies are warranted with a larger number of populations.

## Data Availability

The original contributions presented in the study are included in the article/Supplementary Material, further inquiries can be directed to the corresponding authors.
